# Effects of autonomic nervous system activation on endothelial function in response to acute exercise in hypertensive individuals: study protocol for a randomized double-blind study

**DOI:** 10.1186/s13063-021-05516-x

**Published:** 2021-08-19

**Authors:** Gustavo Waclawovsky, Liliana Fortini Cavalheiro Boll, Salvador Gomes Neto, Maria Claudia Costa Irigoyen, Alexandre M. Lehnen

**Affiliations:** 1 Instituto de Cardiologia do Rio Grande do Sul/Fundação Universitária de Cardiologia, Porto Alegre, Brazil; 2 Unidade de Hipertensão, Instituto do Coração de São Paulo, Universidade do Estado de São Paulo, São Paulo, Brazil

**Keywords:** Endothelium, Autonomic nervous system, Aerobic exercise, Resistance exercise, Systemic arterial hypertension, Randomized clinical trial

## Abstract

**Background:**

Arterial hypertension has a direct association with endothelial dysfunction and major cardiovascular events. There is evidence showing the benefits of aerobic exercise on flow-mediated dilation (FMD) in hypertensive individuals but little is known about the effect of autonomic nervous system (ANS) activation on FMD of the brachial artery in response to different types of exercise in this specific population. This study aims to examine the effects of ANS activation on FMD of the brachial artery in response to exercise in hypertensive individuals following a session of different types of exercise including aerobic exercise (AE), resistance exercise (RE), or combined exercise (CE).

**Methods:**

Thirty-nine hypertensive volunteers aged 35 to 55 years will be randomly assigned to two exercise sessions: AE (40 min on a cycle ergometer at 60% of HR reserve), RE (4 lower limb sets with 12 repetitions at 60% 1-RM for 40 min), or CE (RE for 20 min + AE for 20 min). Each exercise group will be randomized to receive either an α1-adrenergic blocker (doxazosin 0.05 mg/kg^−1^) or placebo. Ultrasound measurement of FMD is performed 10 min before and 10, 40, and 70 min after exercise. ANS activation is monitored using a Finometer and measurements are taken during 10 min before each FMD assessment. Arterial stiffness is assessed by pulse wave velocity (PWV) analysis using a Complior device.

**Discussion:**

We expect to demonstrate the effect of ANS activation on FMD of the brachial artery in hypertensive individuals in response to different types of exercise. This study may give some insight on how to improve exercise prescription for hypertension management.

**Trial registration:**

https://clinicaltrials.gov and ID "NCT04371757". Registered on May 1, 2020.

**Supplementary Information:**

The online version contains supplementary material available at 10.1186/s13063-021-05516-x.

## Introduction

Endothelial dysfunction precedes atherosclerosis [1]. Endothelial cells play an important role in modulating vascular angiogenesis and vascular tone and permeability and mediating inflammatory responses and homeostasis [2]. This function of vascular protection is largely attributed to nitric oxide endothelial synthase, an enzyme responsible for nitric oxide (NO) synthesis in the vascular endothelium [2].

Endothelial dysfunction is characterized by a reduction of local NO bioavailability and/or insufficient vasomotor response that occur in certain conditions including arterial hypertension [3], diabetes mellitus [4], obesity [5], dyslipidemia [6], metabolic syndrome [7], aging [8], menopause [9], and sedentary lifestyle [10]. Flow-mediated dilation (FMD) of the brachial artery is an indirect non-invasive technique to assess endothelial function and is chiefly dependent on *NO availability* [11]. Since a*cute* post-*exercise changes in vascular function* may also predict long-term adaptations [12] the acute response to exercise has been widely studied [3, 13].

Lifestyle changes including regular physical exercise are recommended as part of the therapeutic approach for hypertension management [14, 15]. Factors most commonly associated with potential antihypertensive effects and chronic post-exercise changes in endothelial function include reduced autonomic nervous system (ANS) activation [16], balance between vasodilators and vasoconstrictors [17], and reduced levels of the vasoconstrictor endothelin-1 (ET-1) [18].

A meta-analysis that examined cardiovascular risk factors and/or factors associated to established cardiovascular disease found that aerobic and resistance training can potentially improve FMD of the brachial artery [19]. Our research group also found an improvement of 1.45% (95% CI − 0.11 to 3.00) in FMD in response to aerobic training in hypertensive individuals [20]. This finding is clinically relevant as a 1% increase in FMD is correlated to ~ 13% risk reduction of major cardiovascular outcomes [21].

A study of the effects of resistance exercise (RE) on FMD [22] found that a single session of low- and moderate-intensity knee extensions reduced FMD 10, 30, and 60 min after exercise in healthy individuals. Interestingly, FMD did not change when this same set of RE was performed at a high intensity [22], which may indicate an inverse relationship between FMD changes in response to RE as well as to exercise intensity. Our research group has recently reported that a single session of aerobic exercise (AE), lower-limb RE, and CE (AE + RE) at moderate intensity for 40 min did not result in brachial artery FMD changes in hypertensive individuals when measured 10, 40, and 70 min after exercise [23]. In addition, the levels of endothelial microvesicles and substances associated with oxidative stress remained unchanged [23]. Altogether, we can raise the hypothesis that other factors may play a role including exacerbated sympathetic activation in untrained limbs, which may cause vasoconstriction and redirection of blood flow to trained limbs [24, 25], thus potentially competing with the endothelium-dependent vasodilator effect [26].

Alves et al. [27] demonstrated that α1-adrenergic block with phentolamine reduced blood flow in the forearm when compared to placebo during a handgrip exercise session in individuals with heart failure. It has been proposed that exacerbated sympathetic activation plays an important part in the development and maintenance of arterial hypertension [28], so we postulate that following an acute exercise session sympathetic activation potentiates vasoconstriction and may compete with endothelium-dependent vasodilation effects in hypertensive individuals.

In view of inconsistent findings of the acute effects of different types of exercise on FMD in healthy and hypertensive individuals as well as the scarcity of data of the effects of ANS activation on vascular reactivity in response to exercise in hypertensive individuals, we designed a randomized clinical trial with the primary objective of examining the effects of ANS activation on FMD of the brachial artery in response to acute exercise (AE, lower-limb RE, and CE) in hypertensive individuals. Table 1 summarizes the study objectives.

**Table 1:** Study objectives

**Primary objective**	
• To compare measurements of FMD of the brachial artery in hypertensive individuals receiving either an α1-adrenergic blocker or placebo after sessions of different types of exercise.	
**Secondary objectives**	
• To compare the effects of ANS activation in hypertensive individuals receiving either an α1-adrenergic blocker or placebo (between-group) in response to acute exercise (aerobic, resistance and combined exercise sessions);	
• To correlate the levels of arterial stiffness and ANS activation and FMD of the brachial artery in hypertensive individuals receiving either an α1-adrenergic blocker or placebo in response to acute exercise (aerobic, resistance, and combined exercise sessions).	

*FMD* flow-mediated dilation, *ANS* autonomic nervous system

## Methods/design

### Study setting

This is a randomized, double-blind (blinded to the drug/placebo but not to the type of exercise) parallel-group exploratory clinical trial with a group allocation rate of 70% for the calculated sample. Our study was approved by the local ethics committee (#5678/19) and it will be conducted at Instituto de Cardiologia do Rio Grande do Sul/Fundação Universitária de Cardiologia (ICFUC) – study site. We will use radio, television, social media advertisements to recruit volunteers as well as invitations through phone calls to eligible individuals following a review of medical records from the hospital where the study will be held. All eligible individuals must read and sign a free and informed consent (supplementary material) before entering the study. The study protocol is being developed following the principles of the Declaration of Helsinki. All study information is confidential; volunteers’ names will be kept confidential and all data obtained in the study will be used for academic purposes only. Research data will be stored for five years and then discarded according to the guidelines of CNS Resolution No. 466/12. Finally, any adverse effect during the research will be reported to the local ethics committee that will have access to the adverse results and will make the decision in relation to terminating the study, if applicable. Relevant protocol amendments such as changes to eligibility criteria, unwanted effects, violation of the rights of volunteers, registration, and analysis of results as well as contributions of the study investigators will be reported to the research project coordinator (A.M.L.) and then to the local ethics committee. The study protocol will be updated in the clinical trial registry if applicable.

The study will not receive financial support from any institution, including from ICFUC. Also, ICFUC was not involved in the study design and will not be involved in either data collection, management, analysis, and interpretation or manuscript writing and any decisions regarding submission of articles for publication.

The coordinating center and trial steering committee are tasked with recordkeeping and reviewing ethical issues. The Research Unit (UP/ICFUC) is responsible for registering clinical trials and managing the registration and provides daily support to any issues regarding trial registration. The local ethics committee (EC/ICFUC) is an independent board consisting of academics and representatives of civil society that meet once a month to grant ethical approval for research projects and evaluate research proposals for their importance to society. In addition, research integrity and timeline are overviewed by ICFUC Health Sciences Graduate Program Advisory Committee.

### Trial status

The study is registered on “www.clinicaltrials.gov” (ID: NCT04371757), the anticipated primary completion date is December 20, 2020, and the expected date for full completion of the study (analysis and presentation of results) is December 23, 2021. However, because of the COVID-19 pandemic worsening in Brazil, all clinical research projects have been temporarily suspended at ICFUC (the study site). The expected date for resumption of research activities is November 26, 2021, and the expected completion date is November 25, 2022.

This study protocol has a single version with no updates or addenda since it was registered with ClinicalTrials (“First Posted: May 1, 2020 and Last Update Posted: May 1, 2020”).

### Eligibility criteria

Recruitment will be through on-site screening, media postings, and patient databases. Thus, hypertensive male and female individuals aged 35 to 55 years receiving health care at the study site, using anti-hypertensive drugs, who do not engage in regular physical exercise (≥ 2 sessions per week) are eligible to participate. Exclusion criteria are individuals with diabetes mellitus; chronic renal failure; body mass index (BMI) ≥ 35 kg/m^2^; coronary artery disease; heart failure; users of beta-blockers and/or alpha-blockers; smoking; lower limb injuries that prevent them from engaging in the study intervention; and females aged 35 to 55 with menopause. If doxazosin induces a change in exercise workload or a reduction in blood pressure to levels that would affect exercise performance, the volunteer will be excluded from the study. If a volunteer drops out before completing both study interventions (α1-adrenergic blocker or placebo), we will make an effort to reach them and find out the reasons behind it. If their decision is final, it will be considered “subject withdrawal” and their data will be excluded from the final analyses.

### Sample size

The sample size is calculated according to Atkinson et al. [26]. In their study, they compared endothelium-dependent vasodilation (by FMD) after a single session of moderate-intensity AE for 30 min in healthy individuals receiving an α1-adrenergic blocker (prazosin 0.05 mg/kg^−1^) or placebo and reported a *difference in means of* 3.1% with standard deviations (SDs) of 1.13% for placebo and 1.91% for prazosin. For finding a similar difference in FMD (3.1%) between these two conditions (α1-adrenergic receptor block versus placebo) after our protocol’s exercise session (AE, RE, and CE) considering *α* = 0.05 and 80% power, a sample of five volunteers per group (*n* = 15) is required.

For a comparison of FMD measurements among exercise types (AE, RE, and CE), our sample calculation is based on pre-exercise SD (1.18%) and post-exercise SD (1.91%) for the prazosin group as described by Atkinson et al. Thus, to find a difference in FMD of 1.62% considering *α* = 0.05 and 80% power, a sample of 10 individuals per group (*n* = 30) is required. Assuming a 30% loss, a sample of 39 individuals would be required (*n* = 13 per group).

### Randomization and blinding

Randomization of the study interventions (exercise groups) as well as assignment to intervention conditions (either an α1-adrenergic blocker or placebo) are carried out *using computer program* (www.randomization.com) with a coded numeric distribution. Allocation concealment is guaranteed by using sealed envelopes; the random allocation of volunteers is kept in an inaccessible place and researchers will not have a priori knowledge of the intervention assignment to each volunteer. Randomization is carried out by an independent researcher not involved with the study.

Considering the volunteers’ age group (35 to 55 years) and that comparison analyses (primary and secondary outcomes) involve testing for baseline differences, we are conducting simple randomization divided into two steps: (1) by exercise type (AE, RE, or CE) and (2) the order of condition (either placebo or α1-adrenergic blocker). All volunteers are blinded to the intervention allocation and will only have access to this information after a baseline evaluation. They are also blinded to the intervention condition (either placebo or α1-adrenergic blockade). The researchers responsible for the analyses of data are blinded to the intervention allocation and condition to minimize potential measurement bias. As the medical staff and investigators are not blinded, unblinding will not occur.

### Evaluation and interventions

Volunteers are selected based on information from their medical records. They are first contacted through a telephone call to *ascertain* whether they fit the *inclusion criteria* and *do not meet any* of the *exclusion criteria*. They are then pre-selected and invited to participate in the study. Those who agree to participate are scheduled their first visit (visit 1) at the study site.

**Fig. 1:**
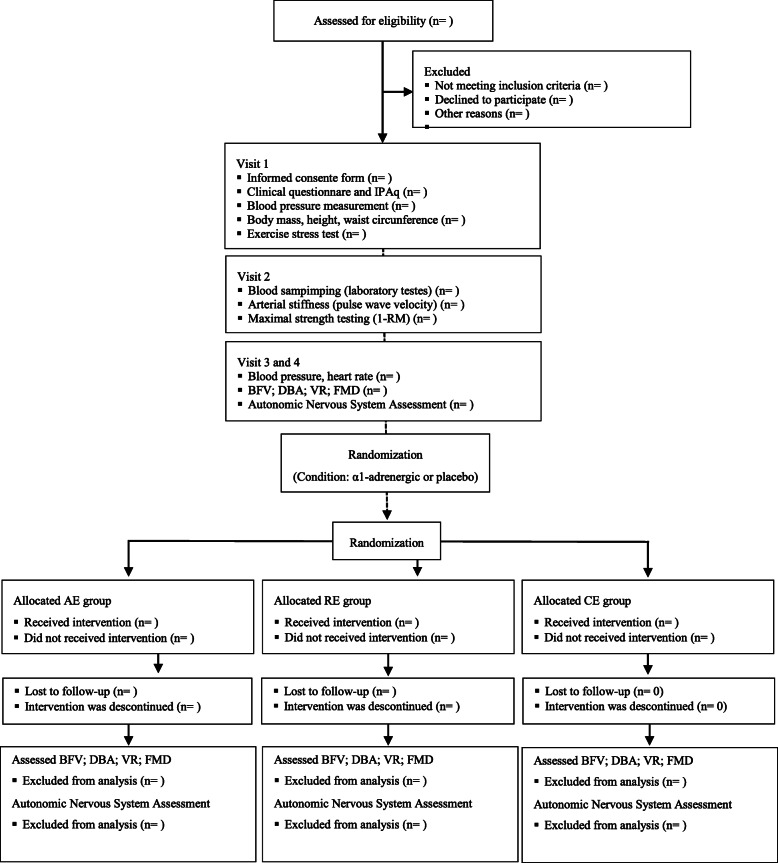
Study design. IPAq: International Physical Activity Questionnaire; AE: aerobic exercise session; RE: resistance exercise session; CE: combined exercise; FMD: flow-mediated dilation; BFV: blood flow velocity; DBA: diameter of brachial artery; VR: vascular resistance

**Fig. 2:**
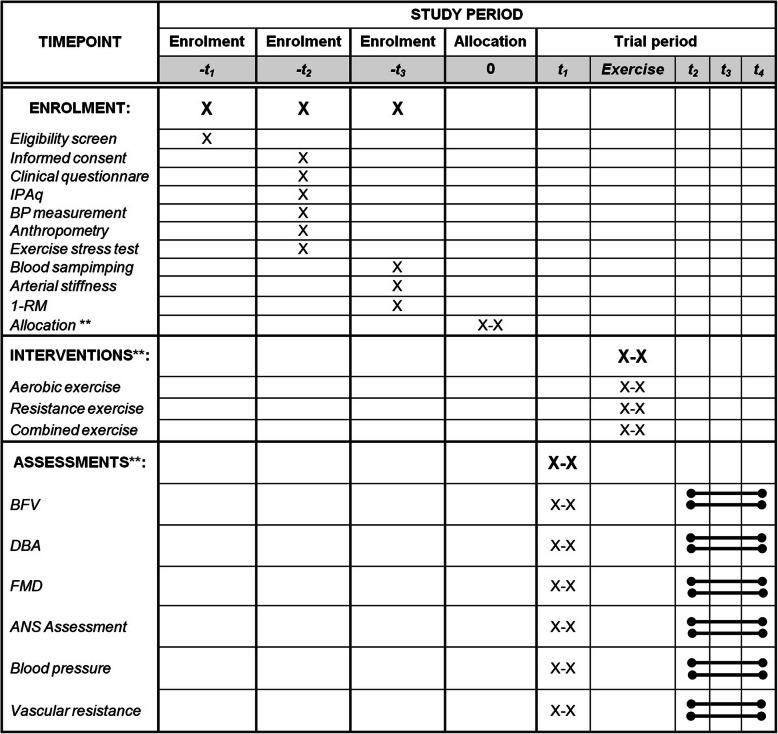
Schedule of enrolment, interventions, and assessment. Adapted from SPIRIT Figure 2013. **Subjects will be allocated to perform twice (X-X) each exercise session on alpha-1 adrenergic block and placebo conditions. IPAq: International Physical Activity Questionnaire; BP: blood pressure; 1-RM: one-repetition maximum; BFV: blood flow velocity; DBA: diameter of brachial artery; FMD: flow-mediated dilation; ANS: autonomic nervous system. Study period: -t1, on-site screening (at Instituto de Cardiologia do Rio Grande do Sul/Fundação Universitária de Cardiologia – ICFUC), media postings, and patient databases; -t2, first visit of the volunteer to the research (ICFUC); -t3, second visit of the volunteer to the research (ICFUC); 0, contact with the independent researcher not involved with the study for volunteer randomization access; t1: pre-exercise assessments (10 min); t2, t3, t4: post-exercise session evaluation (10, 40, 70 min)

**Fig. 3:**
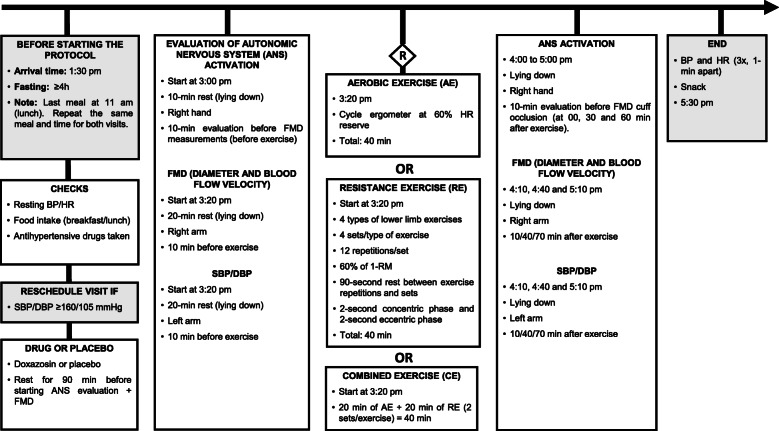
Schedule of intervention day. BP: blood pressure; HR: heart rate; SBP: systolic BP; DBP: diastolic BP; ANS: autonomic nervous system; FMD: flow-mediated dilation; 1-RM: one-repetition maximum

Eligible individuals go through a four-visit protocol as described below. Figure 1 shows the detailed flowchart of the study; Fig. 2 summarizes the recruitment, study interventions, and assessments; and Fig. 3 describes the study intervention (exercise and condition).

Since the study is designed to assess the acute effects of different types of exercise types under the condition of α1-adrenergic blockade or placebo, female volunteers are to undergo evaluations and exercise sessions during the luteal phase of the menstrual cycle. One strategy to improve adherence to the study protocol is to provide the volunteers the results of their laboratory tests and clinical assessments (IPAq, blood pressure and anthropometric measurements, cardiopulmonary test, biochemical analysis, arterial stiffness, and endothelial function assessments and evaluation of ANS) when they complete the study protocol (after their last visit). In case of subject withdrawal, the results will be made available to the volunteers on demand upon study completion.

### Visit 1: interview, anthropometric assessment, and exercise test

Visit 1 takes place at the Laboratory of Clinical Investigation (LCI) at the study site. All inclusion and exclusion criteria are ascertained and the principal investigator (G.W.) will provide the volunteers detailed information on the study and allow time for questions before asking them to sign a free and informed consent form. They will be asked whether they agree to the use of their data should they choose to withdraw from the trial. They will be also asked whether they give permission for the research team to share relevant data with staff from other universities taking part in the research or regulatory authorities, where applicable. This trial does not involve collecting biological specimens for storage. All research data collected will be stored in an appropriate safe space at the study site that is accessible to the principal investigator (G.W.) only.

They are then administered a medical questionnaire the International Physical Activity Questionnaire – long version (IPAQ, http://www.ipaq.ki.se). The volunteer is asked to remain in a seated position in a quiet room for 5 min before their blood pressure (BP) is measured (OMRON™ HEM-714INT device, Tokyo, Japan); three recordings are taken 1 min apart on the arm with the highest values. Those who meet all inclusion criteria undergo an anthropometry assessment and a pre-scheduled exercise stress test.

The anthropometry assessment takes place at the study site. It includes measurements of total body mass (measured with appropriate one-piece [males] and two-piece clothing [females] in a standing position barefoot with feet together at the center of the scale); height (measured in a standing position with feet together, the head in the Frankfort plane with the stadiometer on the top of the head and readings taken while breathing in); waist circumference (measured in a relaxed standing position with arms folded across the chest with the measuring tape placed around the abdomen at the narrowest point between the lower costal margin and the upper part of the iliac crest, the reading is made in the front perpendicular to the longitudinal axis of the body) [29].

The exercise stress test is performed on a treadmill (Inbramed, Porto Alegre, Brazil) using the Bruce Protocol and following the Brazilian Society of Cardiology guidelines [30]. In a testing room at the study site, a qualified technician and a cardiologist conduct the stress test followed with measurements of maximum oxygen consumption, respiratory parameters, and maximum heart rate (HR) (ErgoPC 13, MICRIMED, Brasília, Brazil). Aerobic exercise is to be prescribed according to these measurements. The volunteers are then released by the cardiologist. They are contacted by phone to schedule visit 2.

### Visit 2: blood collection, assessment of arterial stiffness, and maximum strength test

On visit 2 the volunteers are asked to come to the study site after a 12-h overnight fast for blood collection and biochemical analysis (blood count, fasting blood glucose, HbA1c levels, triglycerides, total cholesterol, low-density lipoprotein *cholesterol* [LDL-c], and high-density lipoprotein *cholesterol* [HDL-c], creatinine and ultra-sensitive *polymerase chain reaction* [PCR] assay). LDL-c levels and glomerular filtration rate (GFR) are calculated as detailed below.

- LDL-C = (total cholesterol − HDL-C) − (triglycerides/5);
- GFR (mL/min/1.73 m^2^) = 175 × creatinine^−1.154^ × age^−0.203^ × 1.212 (for blacks) × 0.742 (for females) [31].

Volunteers are then offered a standard snack and asked to rest in a dark, quiet room at 23–24 °C for 10 min. Arterial stiffness is assessed by carotid-femoral pulse wave velocity (PWV) [32] using the Complior Analyse (ALAM Medical, Paris, France). The straight distance between the two arterial sites is measured with an infantometer and data on BP, height, total body mass, and distance between carotid and femoral artery sites are entered into the measuring device software for PWV calculation [32].

The volunteers then undergo the one-repetition maximum (1-RM) test to assess lower limb maximum strength [33] in a proper exercise room at the study site. They first learn the exercise techniques and are instructed on how to perform knee extension, knee flexion, leg pressure, and plantar flexion exercises on a guided weight machine (Movement Perform W8; São Paulo, Brazil). Warm-up consists of 10–12 repetitions, 30% of 1-RM (2 concentric: 2 eccentric phases controlled by a metronome). After a rest of 3–5 min, they start the 1-RM test. It is interrupted to adjust the load when they are able to perform more than one repetition or when they are not able to lift the load. A 5-min rest is allowed before a new attempt is made. The 1-RM is determined when the volunteer performs one complete movement [33]. Their BP is measured 5, 10, and 15 min after the end of the test. The study interventions are scheduled afterwards.

### Visits 3 and 4: α1-adrenergic receptor blockage, assessment of endothelial function, autonomic nervous system activation, and exercise intervention

#### α1-adrenergic receptor blockage

α1-adrenergic blockage is used to assess the effects of sympathetic nervous system (SNS) activation on FMD in response to exercise as described in the literature [26]. Since prazosin is no longer available in Brazil, its analog doxazosin is used in this study [34]. Briefly, 90 min before the start of the exercise intervention, the volunteers are asked to take a tablet containing either placebo (microcrystalline cellulose) or an *α*1-adrenergic blocker (doxazosin 0.05 mg kg^−1^ of body weight) and to lie down and rest. This dose of an *α*1-adrenergic blocker has been reported as being able to block 80% of α1-adrenergic receptors in studies of sympathetic activation and vascular responses [35–37].

An independent researcher blinded to the study intervention gives a tablet to the volunteer on the day of the exercise session according to previous random assignment.

#### Assessment of endothelial function

endothelial function is assessed by FMD as described elsewhere [38, 39]. In a quiet, dark room with controlled temperature (23–24 °C) at the study site, a qualified evaluator blinded to the intervention condition simultaneously measures blood flow velocity and baseline brachial artery diameter on the volunteer’s right arm. With the volunteer in supine position, arm extended and across the trunk at an angle of ~ 40° after a 10–15-min rest, measurements of baseline BP (left arm), resting HR, blood flow velocity, and brachial artery diameter are taken. They are to be evaluated 10 min before and 10, 40, and 70 min after the exercise session.

For the assessment of FMD of the brachial artery, the volunteers are asked to come to the study site after 6-h fasting except for anti-hypertensive medication. A rapid deflation cuff (Incoterm™ EC500; Porto Alegre, Brazil) is placed around the forearm 5 cm distal to the antecubital fossa, and B-mode artery images are obtained at the distal third of the arm with a linear multi-frequency transducer (12 MHz) connected to a high-resolution Doppler ultrasound system (Esaote MyLab™ 70 XVision; Genoa, Italy). The positions of the ultrasound probe and the cuff are marked on the skin so that they can be reproduced during the study. The position of the arm is photographed and the distance between the transducer and the antecubital fossa is measured so that the same can be reproduced in the second exercise session. The evaluator aligns the cursor of the sample volume to the width of the artery at an insonation angle of 60°. Baseline diameters are recorded for 1 min and the forearm cuff is inflated to 50 mmHg above systolic blood pressure (SBP) for 5 min. Images are recorded 30 s before cuff deflation and for 3 min after cuff deflation. Real-time Doppler ultrasound images are recorded and stored into a USB video card (Dazzle; Taiwan) for offline data analysis. To minimize bias blood flow velocity and brachial artery diameter are evaluated via an edge-detection and wall-tracking software program (CardiovasculareSuit™; Pisa, Italy). From this synchronized brachial artery diameter and blood flow velocity data, blood flow (the product of lumen cross-sectional area and Doppler velocity) is calculated at 30 Hz. Forearm peripheral vascular resistance is determined as mean BP divided by blood flow. FMD is calculated as the percentage change in peak diameter after cuff deflation from baseline. Time to peak diameter is calculated from the point of cuff deflation to peak artery diameter [39]. The shear rate is calculated (4 × mean blood velocity/artery diameter) as the area under the curve between the *point* of deflation *and* maximal *dilation* [12].

#### Evaluation of autonomic nervous system activation

ANS activation is evaluated through heart rate variability (HRV) and blood pressure variability (BPV) as described by Atala et al. [40]. Data are collected through continuous non-invasive measurements of pressure waves using digital photoplethysmography (Finometer®, Finapres Medical System BV, Netherland). A cuff device is worn on the middle finger of the right hand coupled to an analog-to-digital converter (AT/MCA-CODAS, DATAC Instruments Inc., Akron, Ohio, USA). Continuous signal sampling acquisition is obtained at 1000 Hz after resting (lying down for 10 min) before FMD. The readings are made for 10 min before forearm cuff inflation for all volunteers either on the placebo or α1-adrenergic blocker groups.

Data are recorded into BeatsScope® (Smart Medical, Gloucestershire, United Kingdom) and LabChart® (ADInstruments, Sydney, Australia) software programs for data analysis of histograms showing BPV and pulse interval series (RR interval) for power spectrum analysis (HRV). All heartbeats are identified through a specialized algorithm using MAT-LAB MT (MATLAB 6.0, Mathworks, USA) with automatic detection of systolic and diastolic pressure wave events. Pulse intervals (PIs) are calculated as the difference between start and end points of the cycle (PI = time point 1 − time point 0). For the time-domain analysis of HRV and BPV data, such as spectrum composition (fast Fourier transform), the CardioSeries® software program is used (www.danielpenteado.com/cardioseries). This analysis requires the removal of transient and discrepant elements and interpolation of consecutive fragments of a time series. The following spectral frequencies are evaluated: very low frequency (VLF, 0.007–0.04 Hz); low frequency (LF, 0.04–0.15 Hz); high frequency (HF, 0.15–0.4 Hz); and total power (TP, VLF + LF + HF). Spontaneous baroreflex sensitivity (SBE) is inferred by the square root of (LF/HRV m^2^)/(LF/BPV m^2^) [40].

#### Reproducibility of FMD

the reproducibility of FMD measurements by the study evaluator (GW) (who has performed more than 150 assessments) was assessed according to current recommendations [38, 39]. Eleven healthy individuals (6 males and 5 females aged 38.4 ± 13.9 years) were tested in the morning (~ 9 h) after a 15-min rest in supine position [38] from October 23 to November 29, 2019. They were evaluated at two time points one hour apart. Reproducibility for each time interval between pairs of values was expressed as the coefficient of variation (CV) of a single measurement, defined as the standard deviation of the difference between paired values divided by the mean and divided by √2) [38, 41]. The mean CV for baseline diameter of healthy volunteers was 1.4 mm and mean CV for FMD was 11.0%. The recommended CV values for diameter (mm) are < 2% and for FMD are < 15% [38].

The exercise sessions are performed in two afternoon visits (1 pm) in random order (placebo or α1-adrenergic blocker). The volunteers are asked to fast over 6 hours before the session. Sessions are held 5 to 7 days apart and include AE on a cycle ergometer; RE; and CE (AE + RE) as described elsewhere [33].

#### Aerobic exercise protocol

AE session is performed on a horizontal cycle ergometer (Movement BM4500 Pro, São Paulo, Brazil) and consists of warm-up (5 min) followed by moderate-intensity AE (60% HR reserve) for 40 min. The volunteers are monitored using a heart rate monitor (POLAR^TM^ RS800CX RUN, Helsinki, Finland) and a subjective effort scale (Borg scores 6 to 20). BP and HR measures and Borg scores are recorded at the start, every 5 min during the session, and up to 15 min after exercise.

#### Resistance exercise protocol

RE consists of lower limb exercise (knee extension, knee flexion, leg pressure, and plantar flexion exercises) on a guided weight machine (Movement Perform W8; São Paulo, Brazil), 4 sets with 12 repetitions, 60% of 1-RM (2 concentric: 2 eccentric phases controlled by a metronome). There is a 90-s rest between sets and exercises and the session lasts 40 min. BP, HR, and Borg scale are recorded at the start and end of set 4 of each exercise as well as up to 15 min after exercise. While performing lower limb strength exercises, the volunteers are not be allowed to hold the hand rests, and an adjustable seat belt is used to stabilize the hip during knee extension and knee flexion [33].

#### Combined exercise protocol

CE consists of AE for 20 min + RE for 20 min (two sets of each exercise) as described elsewhere. Similarly, BP, HR, and Borg scores are recorded at the start and end of RE as well as at the start, every 5 min, and up to 15 min after AE.

#### Additional information

The stress exercise test, 1-RM test ,or exercise session are not started when resting BP is greater than 160 mmHg (SBP) and/or 105 mmHg (DBP) [42]. If BP remains above the recommended levels at a second check the volunteers are referred to the study cardiologist for evaluation. They will be excluded from the study if the cardiologist determines that they cannot participate.

The potential risks and discomforts that volunteers may experience during the experiment include: (i) discomfort due to pressure on their arm when the cuffs are inflated for ultrasound examination (FDM assessments); (ii) discomfort due to pressure on their finger for continuous blood pressure and heart rate monitoring (evaluation of ANS); (iii) “delayed-onset muscle soreness” may occur after exercise; (iv) the blood pressure medication (doxazosin) and its related dose have been used in other studies; however, doxazosin may cause few symptoms including nausea, dizziness, and low blood pressure, which may or may not lead to syncope (fainting).

#### Data management

to ensure data security and data integrity, all research data will be initially collected on printed forms and recorded by each researcher (G.W. and S.G.N.) according to the established procedures. The research project coordinator (A.M.L.) will check the forms for unreadable/illegible entries, inconsistent data and missing information. A collaborator blinded to the study intervention will enter and store data from all forms into the REDCap platform at the study site under the supervision of one of the contributors (L.F.C.B. or M.C.C.I.). Only the authors of this study will have access to it. Each participant has the right of access to their own research results.

After completion of data collection and analysis, relevant data and research findings will be disseminated by preparing research papers for publication in scientific journals with no involvement of professional writers. The dataset will be made available upon publication according to individual journal guidelines.

### Statistical analysis

A per-protocol (PP) analysis will be conducted. The Shapiro-Wilk is used to test the normality of data. Values are presented as means ± standard deviations or medians and 95% confidence intervals (95% CIs) according to the distribution of variables. A multivariate analysis of data is performed using the generalized estimation equations (GEE) for repeated measures with Bonferroni’s post hoc. Pearson’s and Spearman’s tests are used to assess potential correlations between variables according to their distribution. Statistical analyses are carried out using the Statistical Package for Social Sciences (SPSS) 24.0. A level of significance of < 0.05 is set for all analyses.

## Discussion

Regular aerobic exercise has been shown to improve BP [43] and endothelial function [20] in hypertensive patients. Yet there is still scarce evidence on the benefits of resistance training and combined training on BP and endothelial function in hypertensive patients. However, studies have reported improvements in muscle mass and strength [44], as well as bone density [44] in response to these types of exercise supporting their inclusion in routine training. Thus, new scientific evidence on the relationship between exercise and hypertension is still needed particularly on the effects of exercise on FMD modulation in individuals with impaired endothelial function [45].

Exacerbated ANS activation is characteristic of hypertension [28]; this state may compete with endothelium-dependent vasodilation effects and impair vascular response to exercise. This study may help clarify the effects of ANS activation on FMD of the brachial artery in response to different types of exercise in hypertensive individuals. Knowledge of the impact of ANS activation on post-exercise FMD may give some insight on how to improve *exercise prescription* for *hypertension management*.

## Supplementary Information

**Additional file 1.**

## Abbreviations

FMD: Flow-mediated dilation
CV: Coefficient of variance
ANS: Autonomic nervous system
AE: Aerobic exercise
RE: Resistance exercise
CE: Combined exercise
SNS: Sympathetic nervous system
PWV: Pulse wave velocity
NO: Nitric oxide
ET-1: Endothelin-1
FUC: Fundação Universitária de Cardiologia
IC: Instituto de Cardiologia
BMI: Body mass index
GFR: Glomerular filtration rate
LCI: Laboratory of Clinical Investigation
1-RM: One-repetition maximum
HR: Heart rate
BP: Blood pressure
BPV: Blood pressure variability
HRV: Heart rate variability
PI: Pulse interval
VLF: Very low frequency
LF: Low frequency
HF: High frequency
TP: Total power
SBE: Spontaneous baroreflex sensitivity
SBP: Systolic blood pressure
DBP: Diastolic blood pressure
GEE: Generalized estimation equations
