# Supplementary material for: Effects of autonomic nervous system activation on endothelial function in response to acute exercise in hypertensive individuals: study protocol for a randomized double-blind study
Source: Trials. 2021 Aug 19;22:548. doi: 10.1186/s13063-021-05516-x (PMC8374129; doi:10.1186/s13063-021-05516-x)
Supplement: Supplementary file 1 — Additional file 1. [file 13063_2021_5516_MOESM1_ESM.doc]

**FREE AND INFORMED CONSENT FORM**

**Effects of autonomic nervous system activation on endothelial function in response to acute exercise in hypertensive individuals: a randomized double-blind study**

High blood pressure can damage your heart and arteries, yet exercise may reduce the changes caused by this condition. A single exercise session may often not be enough for your arteries to dilate (increase in vessel diameter) or even cause a vasoconstriction response (decrease in vessel diameter), which is not good for people with high blood pressure. This response may be dependent of nerves found in the arteries that can be stimulated during exercise and may affect vasodilation. People with high blood pressure have a more active nervous system in their arteries and this may reduce the beneficial effects (vasodilation) of a session of exercise. However, the effects of the nervous system on the arteries of people with high blood pressure after a session of different types of exercise are not yet known.

This research study will help us assess how much the autonomic nervous system affects the ability of the arteries to dilate in people with high blood pressure after a session of exercise: aerobic exercise (bike); resistance exercise (weight lifting); and combined exercise (bike and weight lifting).

Each participant of this study is asked to follow a four-step protocol:

**Step 1:**questionnaire and anthropometric measurements (weight, height and waist circumference) and an exercise stress test (treadmill test) to determine if you can safely perform the exercise protocol so that we can properly prescribe the session of aerobic exercise. This step is held during week 1 and lasts two hours.

**Step 2:**blood collection after 8-hour overnight fasting for measuring fasting blood glucose, glycated hemoglobin (HbA1c), triglycerides, cholesterol, creatinine and C-reactive protein levels. A snack is offered after blood drawing. Then you are asked to perform a maximum strength test to determine the maximum strength (loads) for different types of exercises (four different leg exercises) so that we can properly prescribe the session of weight lifting exercise. This step is held during week 2 and lasts two hours.

**Steps 3 and 4:**two exercise sessions (assigned by chance) 7 days apart (performed during two similar visits in consecutive weeks): aerobic exercise on an exercise bike for 40 minutes **OR**weight lifting exercise for 40 minutes **OR**a combined exercise session (weight lifting exercise for 20 minutes + aerobic exercise on an exercise bike for 20 minutes). Before each exercise session we examine how rigid your arteries are by placing a sensor on the skin of your neck and upper thigh. Before and after each exercise session we also examine an artery of your arm by ultrasound to see how much it dilates in response to physical exertion. At the same time (before and after exercise) we continuously check your blood pressure and heart rate using a sensor placed on your middle finger. At each visit you are given a capsule that **may or may not contain** a dose of a blood pressure medication (0.05 mg of prazosin for every kilogram of body weight). The capsule dose is adjusted for your body weight; however, you are either given a capsule with the medication or a sugar pill (placebo). For the proper conduct of the research the order of the capsules taken (either prazosin or placebo) is determined by chance. Neither you nor the investigators are aware of which capsule you are given. These steps are held during two visits; each visit lasts 4 hours.

Potential discomfort or risks you may experience are considered to be minimal including discomfort due to the pressure on your arm when the cuffs are inflated for ultrasound examination and due to the pressure on your finger for continuous blood pressure and heart rate monitoring. Delayed muscle soreness may occur after exercise. The blood pressure medication (prazosin) and related dose have been used in other studies. Prazosin may cause some symptoms including nausea, dizziness and low blood pressure, which may or may not lead to syncope (fainting).

If your systolic blood pressure at rest is above 160 mmHg and/or your diastolic blood pressure at rest is above 105 mmHg on the day of the exercise session, you are not able to exercise and a new session is scheduled. If your blood pressure at rest is within the acceptable range but it reaches 220/105 mmHg or above during exercise, the session is discontinued and rescheduled. If your blood pressure remains above the recommended levels you are referred to our cardiologist for evaluation. You can resume the study activities when you are released by the cardiologist.

The exercise tests and sessions are held in our laboratory managed by our team of trained evaluators. There is in our laboratory a proper exercise room equipped for patient monitoring (heart rate and blood pressure) and cardiac events (automatic defibrillator).

Upon completion of all steps of our study protocol you will derive no direct benefits, but you can obtain detailed information about your health including level of physical activity and the results of the exercise stress test, muscle strength test and blood tests. You are reimbursed for travel expenses to the study site.

Your personal information or other health information will be not be shared with others outside this research study. Data will be kept confidential and all information will be used for research purposes only. You have the right to choose to participate or not and your refusal to participate has no impact on health care provided at the Instituto de Cardiologia do Rio Grande do Sul.

By signing this free and informed consent form I state that I agree to participate in this research project. The study objectives and procedures were detailed and stated clearly without any form of constraints and coercion. I was also given assurances that:

- All my questions or any other issues will be addressed about the procedures, risks, benefits and other matters regarding this research project;
- I have the right to withdraw my consent at any time and to end my participation in the study without penalty;
- The results will be reported without revealing my identity and the information provided during this study will be used solely for the purpose of this research project;
- I will be told of any new information that develops during the course of study even if it might affect my willingness to continue participating;
- I agreed that my home phone number may be disclosed so that I can be informed of any developments regarding my health.

I, ________________________________________, hereby state that I have been sufficiently informed about this research study that aims to assess the effects of autonomic nervous system activation on endothelial function in response to acute exercise in hypertensive individuals. My choice to participate in this study has been discussed with the research team. I clearly understand the purposes of the study, procedures to be followed, potential discomfort and risks, assurance of confidentiality and continuing updates. I also understand that my participation does not involve any expenses or remuneration, and that I can withdraw my participation at any time with no penalty or loss of benefits of access and care at this hospital.

**Investigator’s contact information:**If you have new questions about this study or you believe that you may have been harmed in any way, please call the study’s principal investigator Gustavo Waclawovsky any time at (51) 993649958.

You may also call the Research Ethics Committee at Instituto de Cardiologia do Rio Grande do Sul for ethical clarification requests at (51) 32303600 ext. 4136.

You must sign both the original and a copy of this Consent Form. The original signed document is to be retained at the study records and the signed copy is for your records or your legal representative’s records.

Porto Alegre (Brazil), ________________________, 20_____.

_____________________________ _______________________________

Participant’s signature Principal investigator’s signature
